# Prevalence and Risk Factors of Celiac Disease in Patients With Non‐Alcoholic Fatty Liver Disease: A Meta‐Analysis

**DOI:** 10.1002/jgh3.70434

**Published:** 2026-06-22

**Authors:** Narsimha Rao Keetha, Pegah Rashidian, Micah Fleischman, Fatemeh Mohammadyari, Michael T. Ulrich, Seyyed Mohammad Hashemi, Kiran Sandilya Balivada, Mohammad Amin karimi, Negin Letafatkar, Ehsan Amini‐Salehi, Sandeep Samethadka Nayak

**Affiliations:** ^1^ Ohio Kidney and Hypertension Center Middleburg Heights Ohio USA; ^2^ Gastrointestinal and Liver Diseases Research Center Guilan University of Medical Sciences Rasht Iran; ^3^ Department of Physiology and Neurobiology University of Connecticut Storrs Connecticut USA; ^4^ Department of Medicine Riverside University Health System, Medical Center Moreno Valley California USA; ^5^ Cardiovascular Research Center Hormozgan University of Medical Sciences Bandar Abbas Iran; ^6^ Department of Computer Science University of Massachusetts Boston Boston Massachusetts USA; ^7^ School of Medicine Shahid Beheshti University of Medical Sciences Tehran Iran; ^8^ Department of Internal Medicine Yale New Haven Health Bridgeport Hospital Bridgeport Connecticut USA

**Keywords:** celiac disease, meta‐analysis, non‐alcoholic fatty liver disease, prevalence, risk factors

## Abstract

**Background:**

Non‐alcoholic fatty liver disease (NAFLD) has emerged as a significant public health issue due to its close association with metabolic abnormalities. Concurrently, celiac disease (CD), an autoimmune disorder triggered by gluten, has been increasingly investigated in connection with various chronic conditions. Given the potential link between these two conditions, this systematic review and meta‐analysis aimed to determine the prevalence of CD in individuals diagnosed with NAFLD.

**Methods:**

A comprehensive literature search was performed in multiple databases including PubMed, Scopus, Embase, and Web of Science. Studies were considered eligible if they reported clear data regarding CD prevalence among NAFLD populations.

**Results:**

Pooled data analysis revealed an overall CD prevalence of 3.8% (95% CI: 0.02–0.07) among NAFLD patients, with significant heterogeneity observed (*I* ^2^ = 97.36%, *p* < 0.01). Further subgroup analyses demonstrated variations in CD prevalence related to disease characteristics; notably, the prevalence was higher among patients specifically evaluated for CD using serological and histological methods. Exploratory stratified analyses indicated that CD was more prevalent in patients with NAFLD who had lower body mass index (BMI), higher levels of aspartate aminotransferase (AST), lower hemoglobin (HB) concentrations, and reduced platelet (PLT) counts.

**Conclusion:**

The findings from this meta‐analysis suggest a higher‐than‐expected prevalence of CD among patients with NAFLD, though the substantial heterogeneity across studies limits the precision of this estimate. These findings indicate that clinicians may consider selective screening for CD, particularly in patients presenting with specific clinical features. However, given the exploratory nature of the subgroup analyses and the variability in diagnostic approaches across included studies, larger prospective studies with standardized diagnostic criteria are needed to confirm these observations and better elucidate the relationship between these two conditions.

**Abbreviations**
ALP: alkaline phosphatase
ALT: alanine aminotransferase
AST: aspartate aminotransferase
BMI: body mass index
CD: celiac disease
CI: confidence interval
FBS: fasting blood sugar
HB: hemoglobin
JBI: Joanna Briggs Institute
MAFLD: metabolic dysfunction‐associated fatty liver disease
NASH: non‐alcoholic steatohepatitis
OR: Odds Ratio
PLT: platelet
PRISMA: preferred reporting items for systematic reviews and meta‐analyses
TC: total cholesterol
TG: triglycerides

## 1 Introduction

Non‐alcoholic fatty liver disease (NAFLD) celiac disease represents a chronic hepatic disorder increasingly recognized as a critical public health issue due to its widespread prevalence and close linkage with various metabolic abnormalities [1, 2]. This chronic hepatic disease is characterized by hepatic steatosis without significant alcohol consumption, spanning a clinical spectrum ranging from simple steatosis to more severe stages including non‐alcoholic steatohepatitis (NASH), hepatic fibrosis, and potential progression toward cirrhosis and hepatocellular carcinoma in advanced cases [3, 4]. The escalating global prevalence of NAFLD parallels the growing epidemics of obesity, type 2 diabetes mellitus, dyslipidemia, and metabolic syndrome [5, 6]. These metabolic disorders significantly contribute to insulin resistance and chronic low‐grade inflammation, two fundamental mechanisms implicated in NAFLD development and progression [7, 8].

Celiac disease (CD), on the other hand, is a chronic autoimmune condition induced by gluten ingestion in genetically predisposed individuals [9, 10]. It is characterized primarily by inflammatory responses targeting the small intestine, resulting in mucosal damage, malabsorption, and various clinical manifestations, both gastrointestinal and extra‐intestinal [11, 12, 13, 14]. The role of CD in liver pathology has garnered increasing attention, particularly in relation to coexisting conditions such as NAFLD [15, 16]. Emerging evidence has highlighted a potential relationship between CD and hepatic disorders, underscoring the increased susceptibility of CD patients to liver dysfunction. Such associations may be due to mechanisms like immune‐mediated hepatocellular injury, impaired nutritional status, and disturbances in the intestinal microbiome [17, 18, 19, 20]. Collectively, these factors may contribute to disease progression in individuals at risk of developing concurrent CD and liver complications.

Recent evidence has indicated that the prevalence of CD might be elevated among patients with NAFLD in comparison to the general population; however, the exact nature of this association has not yet been fully elucidated [15, 21]. Several potential explanations for this observed link have been proposed, including shared genetic backgrounds, overlapping environmental exposures, and common pathways involving immune system dysregulation [17, 22, 23, 24, 25]. The coexistence of NAFLD and CD in individual patients poses diagnostic and therapeutic challenges, potentially leading to delayed identification or management difficulties. Despite increasing research attention toward understanding the interplay between NAFLD and CD, current literature lacks large‐scale, comprehensive analyses that can definitively assess CD prevalence within the NAFLD population. Consequently, this systematic review and meta‐analysis aims to address this knowledge gap by aggregating and evaluating existing data from multiple studies.

## 2 Methods

The present meta‐analysis was conducted according to the Preferred Reporting Items for Systematic Reviews and Meta‐Analyses (PRISMA) guidelines [26]. The protocol was registered prospectively in the International Prospective Register of Systematic Reviews (PROSPERO) with registration number CRD42025645476. The primary objective of this analysis was to synthesize available data to estimate the prevalence of CD among individuals diagnosed with NAFLD and identify associated factors.

### 2.1 Search Strategy

A thorough and systematic search was conducted in multiple databases—PubMed, Scopus, Embase, and Web of Science—encompassing studies published up until August 18, 2025. No language restrictions were applied. The search strategy included keywords such as “Nonalcoholic Fatty Liver Disease,” “Nonalcoholic Fatty Liver,” “Nonalcoholic Steatohepatitis,” “Celiac Disease,” and “Gluten Enteropathy.” Additionally, the reference lists of significant studies were reviewed to identify further relevant articles. The specific search formulas used for each database are detailed in Table S1.

### 2.2 Study Selection and Eligibility Criteria

The studies included in this meta‐analysis were selected based on clearly defined inclusion and exclusion criteria. Eligible studies needed to meet the following conditions: (a) investigate the prevalence of CD among individuals diagnosed with NAFLD or closely related disorders, such as NASH or metabolic dysfunction‐associated fatty liver disease (MAFLD); (b) report original data on the occurrence of CD in patients with NAFLD or related liver conditions; (c) be published as full‐text articles in peer‐reviewed journals. Studies were excluded if they involved animal models, were available only as abstracts, or evaluated liver diseases unrelated to NAFLD.

### 2.3 Quality Assessment

The quality of the included studies was assessed using the Joanna Briggs Institute (JBI) tool for risk of bias [27, 28, 29]. Any discrepancies between reviewers were resolved through discussion, and the overall quality of evidence was summarized.

### 2.4 Data Extraction

Data extraction for the current meta‐analysis was carried out according to predefined criteria. Information gathered from each study included the following items: the reported prevalence rates of CD among patients with NAFLD, risk factors related to the co‐occurrence of CD and NAFLD, details of patient populations, diagnostic criteria used for both CD and NAFLD, study characteristics, and the statistical methods applied in each study. This process was independently performed by two reviewers, and any discrepancies were settled either by mutual discussion or by seeking the opinion of a third researcher.

### 2.5 Statistical Analyses

Statistical analyses were performed using Comprehensive Meta‐Analysis (CMA) software version 4.0 to synthesize prevalence data across the selected studies. Due to anticipated variability among studies, a random‐effects model was chosen for calculating pooled prevalence estimates. The pooled prevalence of CD was expressed as percentages with corresponding 95% confidence intervals (CIs). Statistical heterogeneity among the studies was assessed using the *I* ^2^ statistic, with values exceeding 50% indicating significant heterogeneity. Sensitivity analyses were subsequently performed by systematically excluding individual studies to evaluate their impact on overall prevalence estimates. Furthermore, prediction intervals were computed to reflect the anticipated range of true prevalence in future research, thereby enhancing the interpretability of the findings. Subgroup analyses were also undertaken to examine the potential sources of heterogeneity and determine whether specific characteristics of the included studies influenced the reported prevalence rates.

## 3 Results

### 3.1 Study Selection

Initially, 962 records were identified through database searches. After removing 479 duplicate records, 483 records were screened. Of these, 462 records were excluded, leaving 21 reports for further assessment.

**FIGURE 1:**
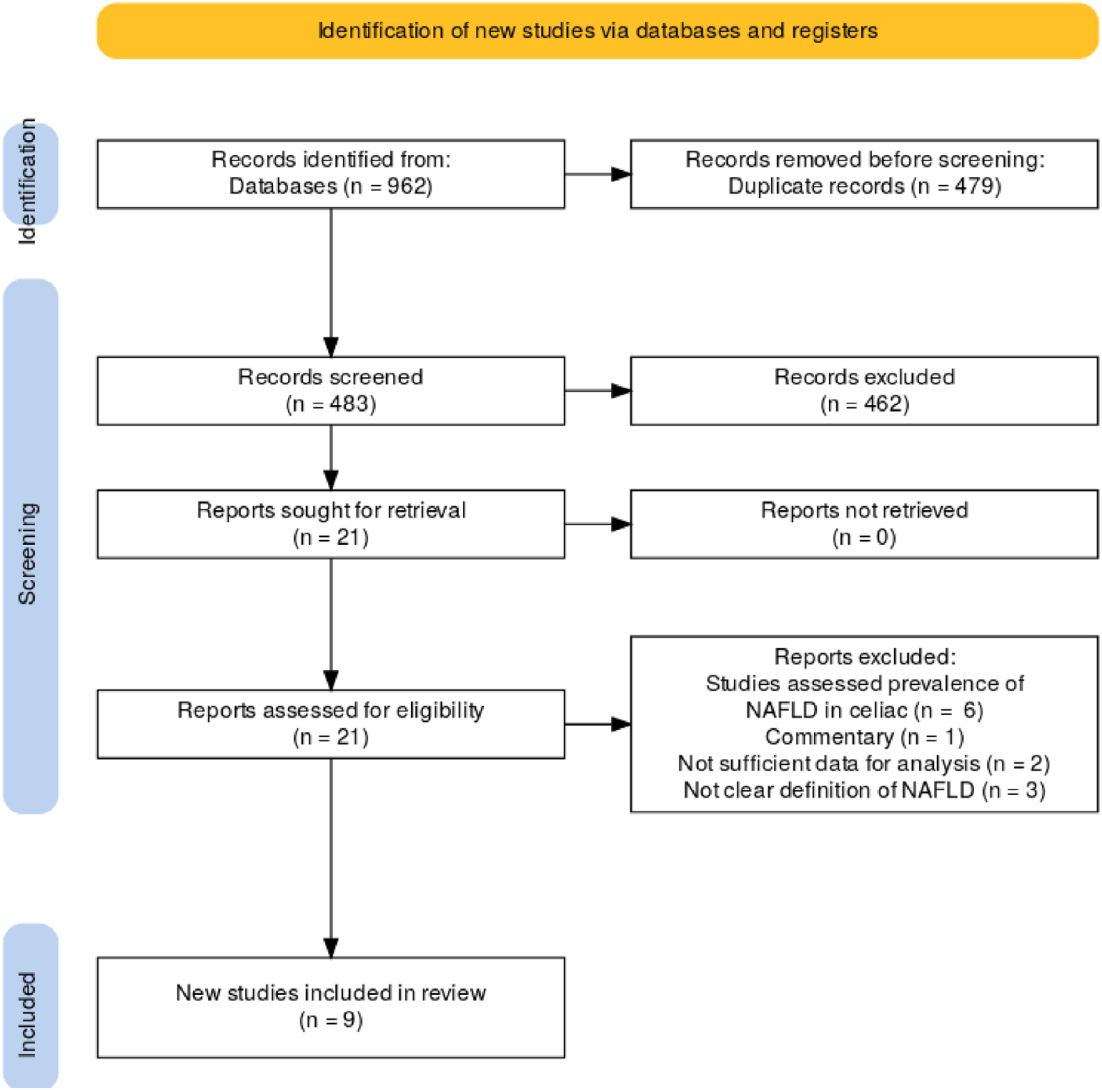
Study selection process.

Out of the 21 reports sought for retrieval, all were successfully retrieved. Upon detailed evaluation, 6 studies were excluded because they assessed the prevalence of NAFLD in CD patients. Additionally, 1 commentary, 2 studies with insufficient data for analysis, and 3 studies with unclear definitions of NAFLD were excluded. Ultimately, 9 studies met the eligibility criteria and were included in the review (Figure 1).

### 3.2 Study Characteristics

The nine eligible studies were conducted in diverse geographical regions, including three from the USA [30, 31, 32], two from Iran [33, 34], and one each from Canada, Italy, Egypt, and the Czech Republic [25, 35, 36, 37]. In 7 studies, intestinal pathology was ultimately used to confirm CD following serological antibody testing [25, 31, 33, 34, 35, 36, 37].

However, one study reported using IgA anti‐endomysial antibody (EMA) as the primary diagnostic tool without confirming the single identified CD case through intestinal pathology [30]. Another study did not clearly specify its diagnostic approach for CD [32].

In terms of study populations, five of the nine studies specifically investigated patients with NASH [30, 31, 32, 35, 36], while the remaining four assessed the prevalence of CD among individuals with NAFLD as a whole [25, 33, 34, 37] (Table 1).

**TABLE 1:** Key characteristics of included studies.

Author, year	Study conduct year	Journal name	Country	Study design	Population	Criteria of celiac	No. of NAFLD/NASH	No. of celiac	Mean age of NAFLD	Mean age of celiac
Bakhshipour, 2013 [33]	2008–2010	Arab Journal of Gastroenterology	Iran	Cross‐sectional	NAFLD	Intestinal pathology	403	13	37.4 ± 12.4	36.61 ± 6.56
Callichurn, 2021 [35]	2013–2016	Journal of the Canadian Association of Gastroenterology	Canada	Cross‐sectional	NASH	Intestinal pathology	113	4	NR	NR
Lo lacono, 2005 [37]	1997–2003	American Journal of Gastroenterology	Italy	Cross‐sectional	NAFLD	Intestinal pathology	121	4	NR	NR
Kamal, 2018 [25]	2011–2016	BMJ Open Gastroenterology	Egypt	Cross‐sectional	NAFLD	Intestinal pathology	2542	182	NR	42.39 ± 7.37
Renno, 2021 [32]	2016–2021	Clinical and Experimental Hepatology	USA	Case–control	NASH	NR	10 950	75	61.21 ± 13.92	NR
Rahimi, 2011 [34]	2008–2009	Turkish Journal of Gastroenterology	Iran	Cross‐sectional	NAFLD	Intestinal pathology	316	7	40.56 ± 11.48	33.57 ± 8.24
Drastich, 2012 [36]	2009–2010	World Journal of Gastroenterology	Czech	Cross‐sectional	NASH	Intestinal pathology	23	1	NR	40
Nehra, 2001 [30]	1996–1997	Digestive Diseases and Sciences	USA	Cross‐sectional	NASH	IgA anti‐endomysial antibody (EMA)	47	1	NR	NR
Wakim‐Fleming, 2014 [31]	2008–2010	Journal of Hepatology	USA	Cross‐sectional	NASH	Intestinal pathology	28	1	NR	62

Abbreviations: NAFLD: non‐alcoholic fatty liver disease; NASH: non‐alcoholic steatohepatitis; NR: not reported.

The risk of bias assessment, summarized in Table S2, indicates that many studies lacked comprehensive data on potential confounding factors and the strategies used to manage them, highlighting a common limitation in this body of research.

### 3.3 Results of Met‐Analysis

#### 3.3.1 Overall Prevalence of CD in Patients With NAFLD/NASH

**FIGURE 2:**
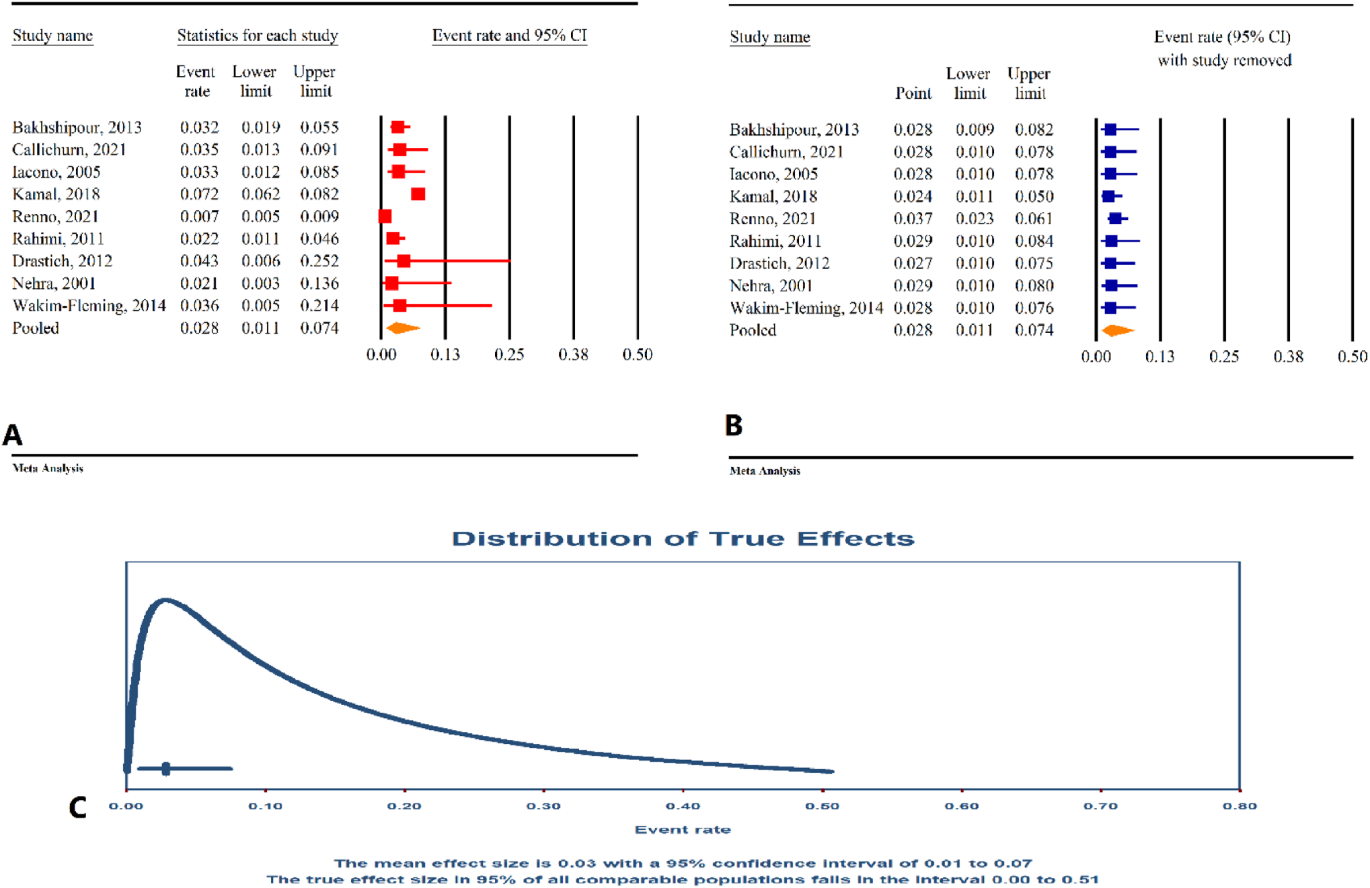
Meta‐analysis of the prevalence of CD in NAFLD/NASH. (A) Forest plot of the overall prevalence of CD in NAFLD/NASH with a 95% CI. (B) Sensitivity analysis demonstrating the impact of individual studies on the overall prevalence estimate. (C) Prediction interval analysis indicating the range within which future studies are expected to fall.

The pooled analysis demonstrated that the overall prevalence of CD among NAFLD/NASH patients was 2.8% (95% CI: 0.01–0.07), as illustrated in Figure 2A. Notably, significant heterogeneity was identified across the included studies (*I* ^2^ = 97.36%, *p* < 0.01). Sensitivity analyses confirmed that none of the individual studies disproportionately influenced the pooled prevalence estimate (Figure 2B). Additionally, the prediction interval was calculated, suggesting that the prevalence of CD in future studies could range from 0.01 to 0.50 (Figure 2C).

#### 3.3.2 CD in Patients With NAFLD and NASH Separately

**FIGURE 3:**
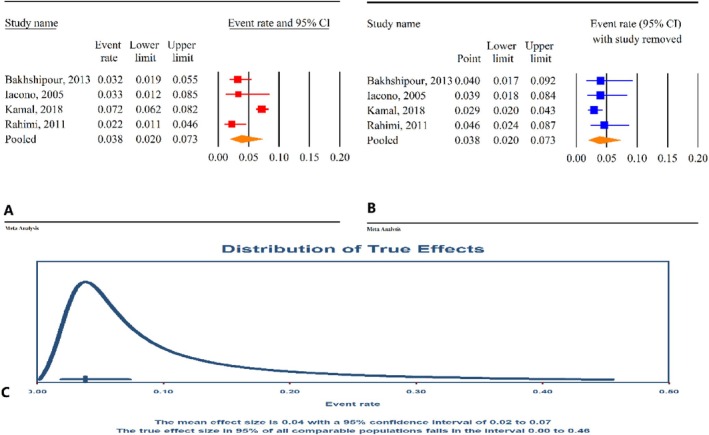
Meta‐analysis of CD prevalence in NAFLD. (A) Forest plot showing the overall prevalence of CD in NAFLD with a 95% CI. (B) Sensitivity analysis demonstrating the impact of individual studies on the overall prevalence estimate. (C) Prediction interval analysis indicating the range within which future studies are expected to fall.

**FIGURE 4:**
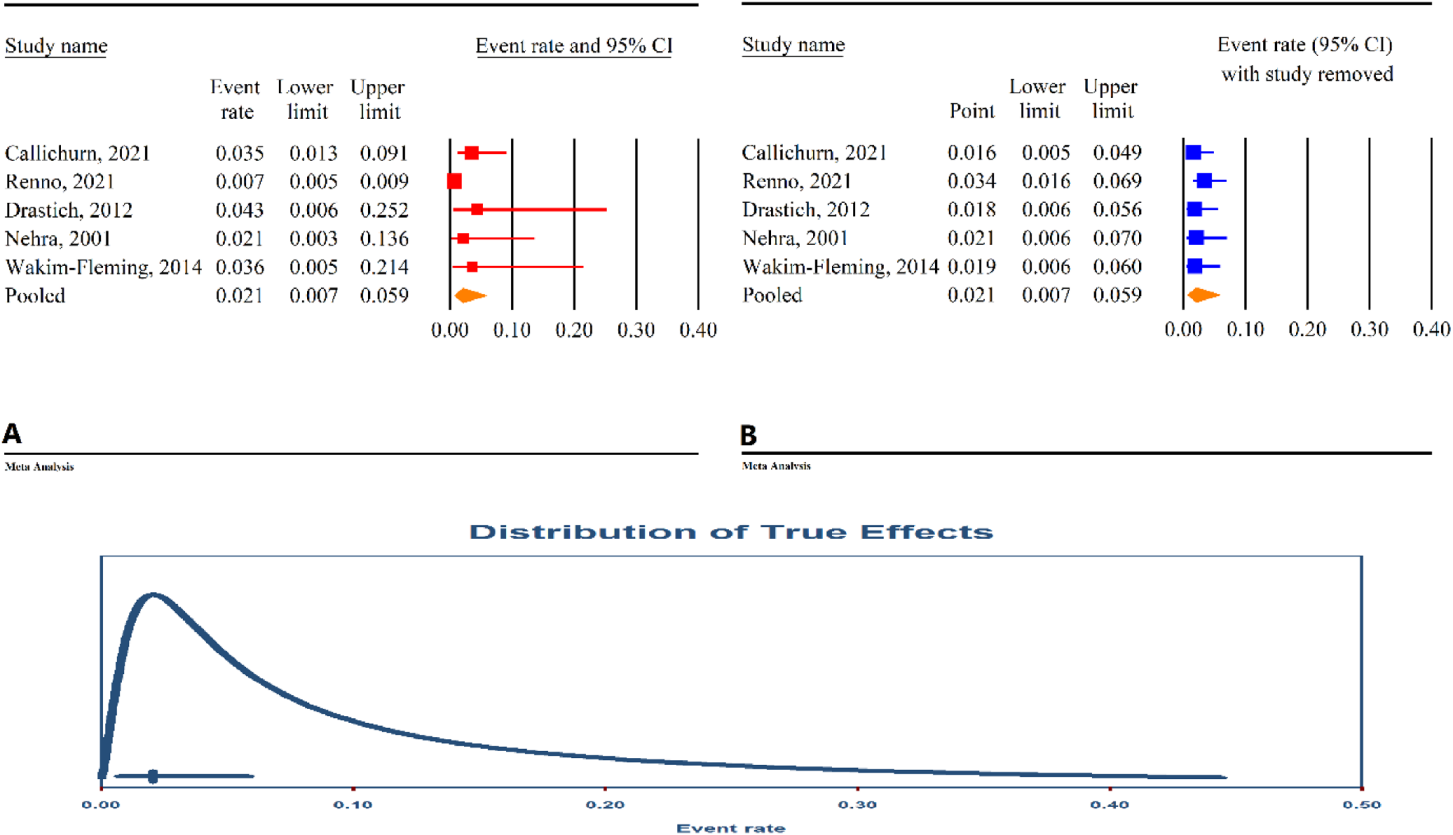
Meta‐analysis of CD prevalence in NASH. (A) Forest plot showing the overall prevalence of CD in NASH with a 95% CI. (B) Sensitivity analysis demonstrating the impact of individual studies on the overall prevalence estimate. (C) Prediction interval analysis indicating the range within which future studies are expected to fall.

The subgroup analysis based on disease type demonstrated that the overall prevalence of CD among individuals with NAFLD was 3.8% (95% CI: 0.02–0.07), while in those with NASH, the prevalence was estimated at 2.1% (95% CI: 0.01–0.05) (Figures 3A and 4A). Heterogeneity analysis indicated substantial variability among the included studies, with an *I* ^2^ value of 84.33% (*p* < 0.01) for NAFLD and 76.06% (*p* < 0.01) for NASH.

Sensitivity analysis was conducted to assess the robustness of the findings, and the results showed no significant impact on the overall estimates following the sequential removal of individual studies (Figures 3B and 4B). Additionally, the prediction interval for the prevalence of CD in NAFLD was estimated to range between 0.01 and 0.45, whereas for NASH, the prediction interval was 0.01–0.44 (Figures 3C and 4C).

#### 3.3.3 Meta‐Analysis of CD Prevalence by Sex

**FIGURE 5:**
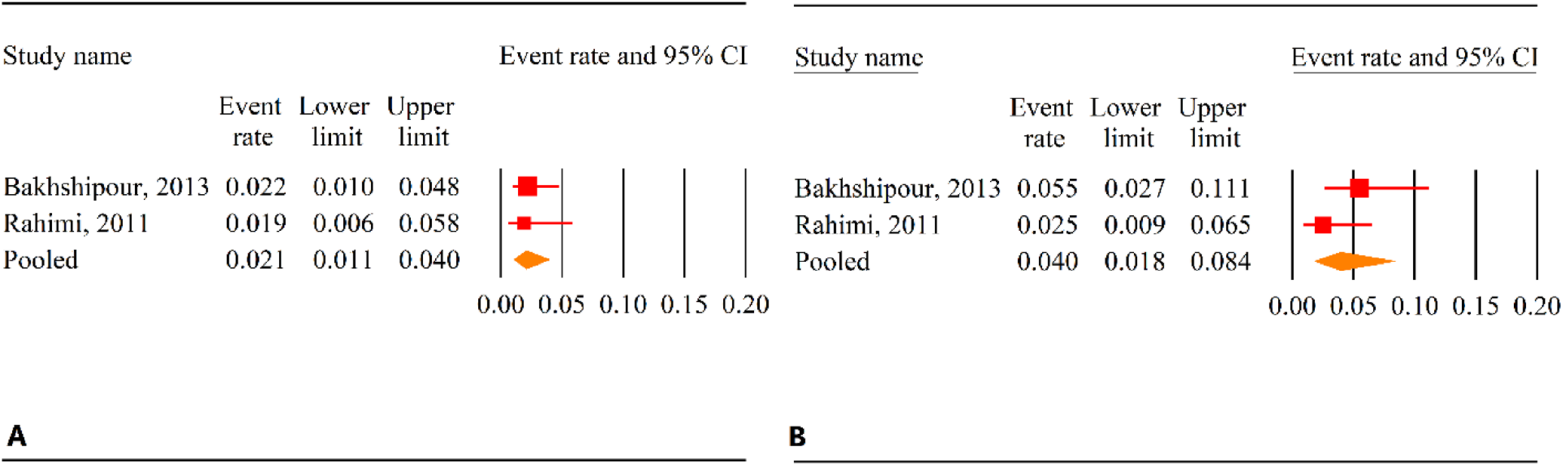
Meta‐analysis of CD prevalence by sex. (A) Forest plot showing the prevalence of CD in men with a 95% CI. (B) Forest plot showing the prevalence of CD in women with a 95% confidence interval.

Exploratory sex‐stratified analysis suggested a numerically higher estimated prevalence of CD among women than men with NAFLD. Specifically, the prevalence was 2.1% (95% CI: 0.01–0.04) among men and 4.0% (95% CI: 0.01–0.08) among women (Figure 5A,B). Heterogeneity was minimal in both subgroups, with *I* ^2^ values of 0.00% (*p* = 0.86) for men and 39.67% (*p* = 0.19) for women. However, due to the limited number of available studies, conducting sensitivity analyses and calculating the prediction interval was not feasible; therefore, this sex‐based difference should be interpreted cautiously.

#### 3.3.4 Celiac Prevalence in Patients With NAFLD/NASH Regarding MARSH Category

**FIGURE 6:**
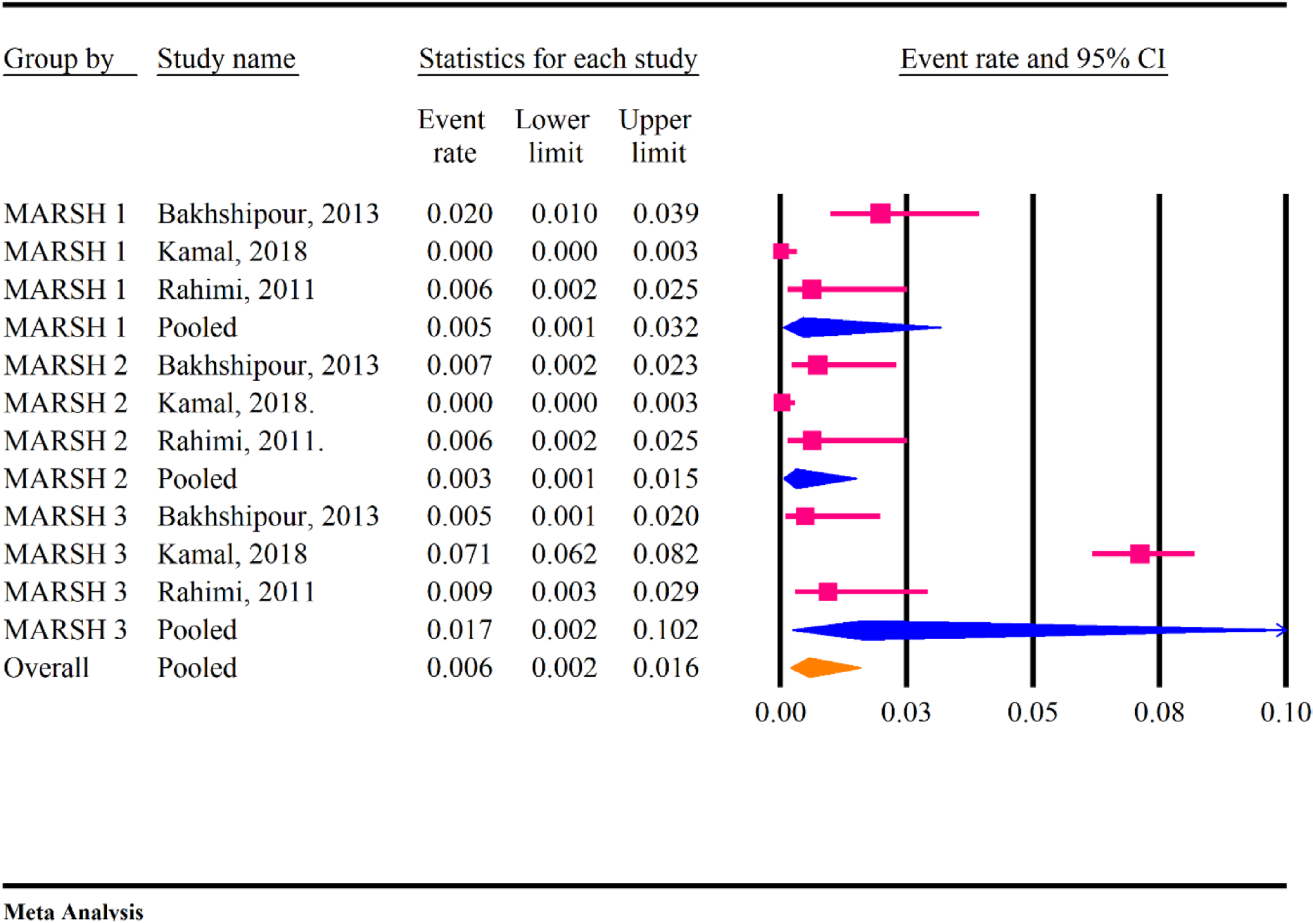
Meta‐analysis of CD prevalence by Marsh Classification in NAFLD/NASH.

Exploratory analysis by Marsh category suggested that, among patients with NAFLD or NASH, CD‐related histological findings were most frequently classified as Marsh 3, with a prevalence of 1.7% (95% CI: 0.002–0.102). This was followed by Marsh 1, with a prevalence of 0.5% (95% CI: 0.001–0.032), and Marsh 2, with a prevalence of 0.3% (95% CI: 0.001–0.015) (Figure 6). Heterogeneity was high for Marsh 1 (*I* ^2^ = 82.35%, *p* < 0.01), Marsh 2 (*I* ^2^ = 71.19%, *p* = 0.03), and Marsh 3 (*I* ^2^ = 92.57%, *p* < 0.01), and these subgroup findings should therefore be interpreted cautiously.

### 3.4 Results of Sub Group Analysis

In exploratory subgroup analyses based on a limited number of contributing studies, several clinical parameters showed statistically significant associations with CD in individuals with NAFLD. Lower BMI may be associated with the presence of CD (OR: 0.12; 95% CI: 0.02–0.58; *p* < 0.01), although this finding should be interpreted cautiously given the limited evidence base and heterogeneity.

Additionally, elevated AST levels may be associated with CD (OR = 2.18; 95% CI: 1.67–2.86; *p* < 0.01), suggesting that individuals with CD and NAFLD tend to have higher AST concentrations. An inverse relationship was also observed between HB levels and CD (OR = 0.05; 95% CI: 0.01–0.20; *p* < 0.01), suggesting that lower HB levels may be more frequently observed among affected individuals.

Similarly, a lower PLT count may be associated with CD in patients with NAFLD (OR = 0.59; 95% CI: 0.45–0.78; *p* < 0.01), suggesting an association between decreased PLT levels and the presence of CD in patients with NAFLD. These statistical findings are summarized in Table 2 and should be regarded as hypothesis‐generating rather than definitive.

**TABLE 2:** Association between clinical factors and CD among patients with NAFLD/NASH.

Variable	Number of studies	OR	95% CI	*p* value	Heterogeneity	*p* value of heterogeneity
BMI	2	0.12	0.02–0.58	< 0.01	79.75%	0.02
AST	3	2.18	1.67–2.86	< 0.01	0.00%	0.52
ALT	3	3.26	0.87–12.18	0.07	85.87%	< 0.01
ALP	2	0.88	0.39–1.97	0.76	0.00%	0.36
TG	3	0.31	0.03–2.69	0.28	94.76%	< 0.01
TC	2	1.66	0.23–11.77	0.61	87.59%	< 0.01
FBS	2	0.59	0.26–1.34	0.21	0.00%	0.78
HB	2	0.05	0.01–0.20	< 0.01	72.89%	0.05
PLT	2	0.59	0.45–0.78	< 0.01	0.00%	0.49

Abbreviations: ALP: alkaline phosphatase; ALT: alanine aminotransferase; AST: aspartate aminotransferase; BMI: body mass index; FBS: fasting blood sugar; HB: hemoglobin; PLT: platelets; TC: total cholesterol; TG: triglycerides.

## 4 Discussion

**FIGURE 7:**
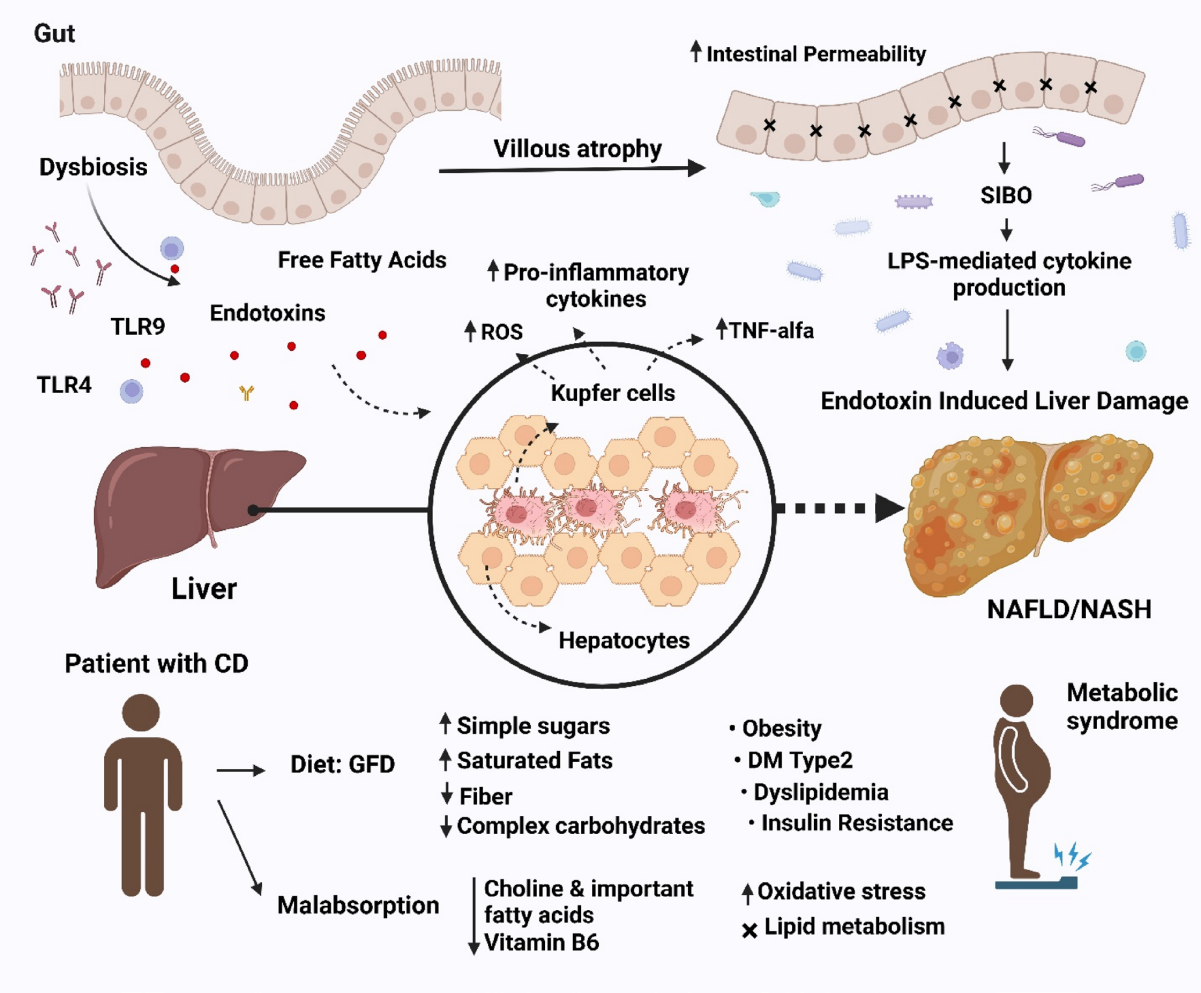
Proposed mechanisms linking CD and NAFLD/NASH. CD: celiac disease; GFD: gluten‐free diet; LPS: lipopolysaccharide; NAFLD: non‐alcoholic fatty liver disease; NASH: non‐alcoholic steatohepatitis; ROS: reactive oxygen species; SIBO: small intestinal bacterial overgrowth; TLR: toll‐like receptor.

The current review included 9 studies verifying the existence of an association between CD and NAFLD, discussing any evidence and conflicting results between supporting and negating it. This study estimates the prevalence of CD among NAFLD patients to be about 3.8%, which is higher than that in the general population, mostly ranging between 0.7% and 2.9% reported across different regions [38]. This finding is consistent with several other studies that have suggested a heightened prevalence of CD in NAFLD populations. For instance, Bakhshipour et al. [33] observed a similar trend in their cohort, reporting a CD prevalence of 3.22% among NAFLD patients. Their study also found that patients with a BMI of less than 25 kg/m^2^ had a markedly higher prevalence of CD, about 15.51%. This reinforces the notion that BMI may be a crucial factor affecting the coexistence of CD and NAFLD, aligning with our study, which demonstrated that NAFLD patients with a lower BMI exhibited greater rates of CD. Additionally, a Swedish countrywide study involving over 26 000 individuals with CD revealed an elevated risk of NAFLD in both children and adults with the condition [15]. The studies conducted by Bardella et al. [22] and Lo Iacono et al. [39] reported a CD prevalence of 3.4% among NAFLD patients with persistent hypertransaminasemia, which is more than six times higher than its prevalence in the general population. Additionally, CD was identified in 3.3% of NAFLD patients with a BMI below 30 kg/m^2^, further supporting the findings of our exploratory analyses. Consistently, our analysis also suggested that elevated AST levels, lower HB concentrations, and decreased PLT counts are significant risk factors for CD in individuals with NAFLD. However, these subgroup findings were based on only a small number of studies and should be interpreted cautiously. Moreover, as suggested in previous literature, CD prevalence differs by sex [40], and our results suggested higher susceptibility among female patients. Recently, in another study by Rahimi et al. [34] prevalence of CD has been reported to be 2.2% in patients with NAFLD; which was 5.8% in patients with BMI value < 27 kg/m^2^. In their study, slight female dominance is compatible with ours. Albeit, their data corroborate that not all patients with CD exhibit normal or low BMI or waist circumference [41], as 2 of 7 individuals (29%) do. They found that transaminase levels did not exhibit a significant difference between CD and non‐CD patients; however, HB levels in CD patients were significantly lower than those in non‐CD individuals, still supporting our notion. Similar to our research, Rahimi et al. [34] also noted that CD should be included in the differential diagnosis for patients with NAFLD. Nevertheless, not all research has identified a robust correlation between CD and NAFLD. For example, Nehra et al. [30] showed no significant association between NASH and CD, noting only one instance of CD among a group of 47 NASH patients. This disparity may be attributed to variations in the study populations, patient demographics, research methodologies, and diagnostic standards. The limited sample size in their study and the lack of a comprehensive examination of variables such as BMI may account for the failure to establish a stronger correlation between the two situations. Foremost, their study's emphasis on NASH, as opposed to a wider spectrum of NAFLD phenotypes, may have influenced the disagreements in findings. In contrast, Kamal et al. [25] found a substantial correlation between CD and NAFLD, especially for the advancement of liver damage. Altogether, most research underscores the imperative for clinicians to contemplate the possible coexistence of CD in patients with NAFLD, particularly in individuals with a lower BMI, in women, and also from traditional malabsorption symptoms, prompting the active screening of celiac antibodies and facilitating early diagnosis. To achieve this goal, being aware of the underlying mechanisms of the association between these two disorders is necessary. This correlation is likely driven by multiple interrelated mechanisms such as gut microbiota dysbiosis [42], endotoxemia [32], increased intestinal permeability [42, 43], metabolic alterations [44], nutritional deficiencies [45, 46], and small intestinal bacterial overgrowth (SIBO) [47] which are discussed respectively (Figure 7).

### 4.1 Gut Microbiota Dysbiosis and Endotoxemia

An increasing amount of evidence indicates that changes in the gut microbiota (dysbiosis) significantly contribute to the pathophysiology of liver disease in CD [42]. Imbalances in gut microbiota among CD patients elevate the influx of fatty acids and toll‐like receptor (TLR) agonists, particularly TLR4 and TLR9, into the liver through the portal circulation. This mechanism stimulates hepatic Kupffer cells, resulting in elevated TNF‐α production, a key mediator of hepatic inflammation and fibrosis [48]. Wigg et al. [47] bolstered this concept by suggesting that gut‐derived endotoxins migrate to the liver, activating Kupffer cells and triggering a cascade of pro‐inflammatory cytokines and reactive oxygen species (ROS). This mechanism closely resembles the etiology of NASH, wherein persistent inflammation and oxidative stress induce hepatic injury and fibrosis. Renno et al. [32] also highlighted the two‐hit concept, wherein intestinal dysbiosis and endotoxemia serve as a secondary component that exacerbates NAFLD progression in CD patients who have previously manifested hepatic steatosis due to metabolic causes [42, 49] (Figure 7).

### 4.2 Increased Intestinal Permeability and the Gut‐Liver Axis

A characteristic aspect of CD is the breakdown of the intestinal barrier, resulting in heightened permeability. This facilitates the translocation of antigens, toxins, and inflammatory mediators into the portal circulation, thereby reaching the liver and leading to hepatic inflammation and damage [42, 43]. Enhanced intestinal permeability in CD may promote the uptake of food and microbial antigens, resulting in immunological activation in the liver [43]. The extent of villous atrophy in CD correlates with intestinal permeability, indicating that untreated CD patients face an elevated risk of endotoxin‐induced liver damage [47, 50, 51]. Meanwhile, Rahimi et al. [34] underscored that intestinal permeability is a pivotal element in the malfunctioning of the gut‐liver axis (Figure 7).

### 4.3 Metabolic Dysregulation and Dietary Influences

CD‐related metabolic alterations, particularly those affecting glucose and lipid metabolism, may further increase the risk of hepatic steatosis and inflammation. Unlike non‐CD patients, where insulin resistance plays a primary role in NAFLD pathogenesis, CD patients may develop NAFLD due to gut microbiota‐induced metabolic disturbances [44, 52]. Dietary habits of CD patients, especially those adhering to a gluten‐free diet (GFD), may predispose them to NAFLD. Studies indicate that CD patients on a GFD consume higher amounts of simple sugars, saturated fats, and processed foods, while their intake of fiber and complex carbohydrates is significantly reduced [16, 53, 54]. These dietary shifts can contribute to obesity, dyslipidemia, insulin resistance, and metabolic syndrome, all of which are risk factors for NAFLD [55]. Additionally, the compensatory hyperphagic state observed in newly diagnosed CD patients may lead to excessive caloric intake, further promoting hepatic lipid accumulation and inflammation [56] (Figure 7).

### 4.4 Nutritional Deficiencies and Oxidative Stress

CD is frequently linked to malabsorption disorders, resulting in deficits of pyridoxine (vitamin B6), choline, and important fatty acids. These nutrients are essential for hepatic lipid metabolism and antioxidative defense systems; their shortage may lead to oxidative stress and hepatic damage [45]. Additionally, free fatty acids accumulate in patients with CD and liver disease, resulting in mitochondrial dysfunction, heightened lysosomal fragility, and compromised membrane integrity [46, 57]. This process causes the production of ROS, lipid peroxidation, and the activation of stellate cells, which are pivotal to fibrosis and hepatocyte death [46, 57, 58, 59, 60, 61]. Rahimi et al. [34] further corroborated the gut‐liver axis concept, indicating that malabsorption and chronic inflammation in CD heighten vulnerability to hepatic oxidative stress and fibrosis, irrespective of conventional metabolic risk factors [43] (Figure 7).

### 4.5 SIBO

The role of SIBO in CD‐related liver dysfunction has gained increasing attention. SIBO is more common in people with CD and is associated with heightened gut permeability, chronic inflammation, and systemic endotoxemia, all of which can contribute to NAFLD progression by inducing LPS‐mediated cytokine production, especially TNF‐α, which facilitates hepatic inflammation and oxidative stress [47, 50, 51, 62]. It was shown that individuals with CD frequently demonstrate SIBO and elevated endotoxemia, factors that exacerbate the severity and progression of NAFLD [43] (Figure 7).

## 5 Limitations and Future Directions

This meta‐analysis has several limitations that should be acknowledged. A primary concern is the heterogeneity observed, which may be attributed to multiple factors including variations in study populations (age, sex, ethnicity, genetic predisposition), geographical and environmental differences, and variability in healthcare access. Additionally, the reliance on retrospective and cross‐sectional designs limits the ability to establish causality between CD and NAFLD/NASH.

The diagnostic approach for CD varied across included studies. While seven studies utilized intestinal pathology to confirm CD following serological testing, one study relied solely on IgA anti‐endomysial antibody without histological confirmation, and another did not clearly specify the diagnostic approach. This variability likely contributed to heterogeneity and affected prevalence estimates.

Furthermore, subgroup analyses examining clinical parameters were based on a limited number of studies. These findings should therefore be interpreted as exploratory observations rather than definitive associations. The lack of uniform reporting on potential confounders, including dietary habits, genetic predispositions, and lifestyle factors, may have further influenced the observed associations. Due to the limited number of available studies, comprehensive subgroup analyses to fully evaluate sources of heterogeneity could not be performed.

We also acknowledge that the terminology used in this manuscript (NAFLD/NASH) reflects the nomenclature employed by the included studies at the time of their conduct. Current consensus recommendations now use the updated terminology MASLD (metabolic dysfunction‐associated steatotic liver disease). Future studies adopting this standardized nomenclature will facilitate more consistent reporting and comparability across the literature.

Future research should focus on large‐scale, prospective cohort studies with standardized diagnostic criteria for both conditions to better understand the causal relationship between CD and NAFLD/NASH. There is also a need for studies that explore the underlying immunological and metabolic mechanisms linking these conditions, which may provide insights into targeted therapeutic interventions. Furthermore, investigating the potential impact of a gluten‐free diet on liver health in NAFLD/NASH patients with CD could offer valuable implications for disease management.

## 6 Conclusion

This meta‐analysis suggests a potential association between NAFLD and CD, with a higher estimated prevalence of CD among NAFLD patients than expected from general‐population estimates. However, substantial heterogeneity across studies limits the reliability and generalizability of the pooled prevalence estimate; therefore, these findings should be interpreted with caution.

The results also suggest that selective CD screening may be considered in specific NAFLD subgroups, particularly in patients with lower BMI, unexplained anemia, thrombocytopenia, elevated AST, or suggestive clinical features. Given the small number of studies contributing to these subgroup analyses, these observations should be considered exploratory and hypothesis‐generating rather than definitive.

Rather than supporting routine integration of CD screening into the clinical management of all NAFLD patients, the present findings suggest that a selective screening approach may help identify patients with underlying CD who warrant further diagnostic evaluation. Future large‐scale, prospective cohort studies with standardized diagnostic criteria for both conditions are essential to establish causality, clarify underlying mechanisms, and evaluate whether diagnosis and treatment of coexisting CD influences NAFLD progression.

## Ethics Statement

The authors have nothing to report.

## Consent

The authors have nothing to report.

## Conflicts of Interest

The authors declare no conflicts of interest.

## Supporting information


**Table S1:** Provides the complete search strategies used across all databases.
**Table S2:** Summarizes the risk‐of‐bias assessment of the included studies according to the Joanna Briggs Institute (JBI) criteria.

## Data Availability

The data that support the findings of this study are available from the corresponding author upon reasonable request.

## References

[1] E. Amini‐Salehi , N. Letafatkar , N. Norouzi , et al., “Global Prevalence of Nonalcoholic Fatty Liver Disease: An Updated Review Meta‐Analysis Comprising a Population of 78 Million From 38 Countries,” Archives of Medical Research 55, no. 6 (2024): 103043. 39094335 10.1016/j.arcmed.2024.103043

[2] S. Pouwels , N. Sakran , Y. Graham , et al., “Non‐Alcoholic Fatty Liver Disease (NAFLD): A Review of Pathophysiology, Clinical Management and Effects of Weight Loss,” BMC Endocrine Disorders 22, no. 1 (2022): 63. 35287643 10.1186/s12902-022-00980-1 PMC8919523

[3] E. Dhamija , S. B. Paul , and S. Kedia , “Non‐Alcoholic Fatty Liver Disease Associated With Hepatocellular Carcinoma: An Increasing Concern,” Indian Journal of Medical Research 149, no. 1 (2019): 9–17. 31115369 10.4103/ijmr.IJMR_1456_17 PMC6507546

[4] Ó. Soto‐Angona , G. Anmella , M. J. Valdés‐Florido , et al., “Non‐Alcoholic Fatty Liver Disease (NAFLD) as a Neglected Metabolic Companion of Psychiatric Disorders: Common Pathways and Future Approaches,” BMC Medicine 18, no. 1 (2020): 261. 32998725 10.1186/s12916-020-01713-8 PMC7528270

[5] F. Radu , C. G. Potcovaru , T. Salmen , P. V. Filip , C. Pop , and C. Fierbințeanu‐Braticievici , “The Link Between NAFLD and Metabolic Syndrome,” Diagnostics (Basel) 13, no. 4 (2023): 614. 36832102 10.3390/diagnostics13040614 PMC9955701

[6] M. de Vries , J. Westerink , K. Kaasjager , and H. W. de Valk , “Prevalence of Nonalcoholic Fatty Liver Disease (NAFLD) in Patients With Type 1 Diabetes Mellitus: A Systematic Review and Meta‐Analysis,” Journal of Clinical Endocrinology and Metabolism 105, no. 12 (2020): 3842–3853. 32827432 10.1210/clinem/dgaa575 PMC7526735

[7] J. P. Nogueira and K. Cusi , “Role of Insulin Resistance in the Development of Nonalcoholic Fatty Liver Disease in People With Type 2 Diabetes: From Bench to Patient Care,” Diabetes Spectrum: A Publication of the American Diabetes Association 37, no. 1 (2024): 20–28. 38385099 10.2337/dsi23-0013 PMC10877218

[8] R. Palma , A. Pronio , M. Romeo , et al., “The Role of Insulin Resistance in Fueling NAFLD Pathogenesis: From Molecular Mechanisms to Clinical Implications,” Journal of Clinical Medicine 11, no. 13 (2022): 3649. 35806934 10.3390/jcm11133649 PMC9267803

[9] G. Caio , U. Volta , A. Sapone , et al., “Celiac Disease: A Comprehensive Current Review,” BMC Medicine 17, no. 1 (2019): 142. 31331324 10.1186/s12916-019-1380-z PMC6647104

[10] G. D. Cazac , B. M. Mihai , G. Ștefănescu , et al., “Celiac Disease, Gluten‐Free Diet and Metabolic Dysfunction‐Associated Steatotic Liver Disease,” Nutrients 16, no. 13 (2024): 2008. 38999756 10.3390/nu16132008 PMC11243569

[11] A. E. V. Tutturen , S. Dørum , T. Clancy , et al., “Characterization of the Small Intestinal Lesion in Celiac Disease by Label‐Free Quantitative Mass Spectrometry,” American Journal of Pathology 188, no. 7 (2018): 1563–1579. 29684362 10.1016/j.ajpath.2018.03.017

[12] M. Durazzo , A. Ferro , I. Brascugli , S. Mattivi , S. Fagoonee , and R. Pellicano , “Extra‐Intestinal Manifestations of Celiac Disease: What Should we Know in 2022?,” Journal of Clinical Medicine 11, no. 1 (2022): 258. 35011999 10.3390/jcm11010258 PMC8746138

[13] A. Santonicola , H. Wieser , C. Gizzi , C. Soldaini , and C. Ciacci , “Associations Between Celiac Disease, Extra‐Gastrointestinal Manifestations, and Gluten‐Free Diet: A Narrative Overview,” Nutrients 16, no. 12 (2024): 1814. 38931169 10.3390/nu16121814 PMC11206979

[14] S. Jabeen , A. U. Khan , W. Ahmed , et al., “Disease Specific Symptoms Indices in Patients With Celiac Disease‐A Hardly Recognised Entity,” Frontiers in Nutrition 9 (2022): 944449. 36159486 10.3389/fnut.2022.944449 PMC9494589

[15] N. R. Reilly , B. Lebwohl , R. Hultcrantz , P. H. Green , and J. F. Ludvigsson , “Increased Risk of Non‐Alcoholic Fatty Liver Disease After Diagnosis of Celiac Disease,” Journal of Hepatology 62, no. 6 (2015): 1405–1411. 25617505 10.1016/j.jhep.2015.01.013 PMC4439270

[16] A. Raiteri , A. Granito , C. Faggiano , et al., “Hepatic Steatosis in Patients With Celiac Disease: The Role of Packaged Gluten‐Free Foods,” Nutrients 14, no. 14 (2022): 2942. 35889899 10.3390/nu14142942 PMC9316041

[17] I. Hoffmanová , D. Sánchez , L. Tučková , and H. Tlaskalová‐Hogenová , “Celiac Disease and Liver Disorders: From Putative Pathogenesis to Clinical Implications,” Nutrients 10, no. 7 (2018): 892. 30002342 10.3390/nu10070892 PMC6073476

[18] F. Pecora , F. Persico , P. Gismondi , et al., “Gut Microbiota in Celiac Disease: Is There Any Role for Probiotics?,” Frontiers in Immunology 11 (2020): 957. 32499787 10.3389/fimmu.2020.00957 PMC7243837

[19] R. E. Rossi , G. Dispinzieri , A. Elvevi , and S. Massironi , “Interaction Between Gut Microbiota and Celiac Disease: From Pathogenesis to Treatment,” Cells 12, no. 6 (2023): 823. 36980164 10.3390/cells12060823 PMC10047417

[20] M.‐H. Keivanlou , E. Amini‐Salehi , N. Sattari , et al., “Gut Microbiota Interventions in Type 2 Diabetes Mellitus: An Umbrella Review of Glycemic Indices,” Diabetes and Metabolic Syndrome: Clinical Research and Reviews 18, no. 8 (2024): 103110. 10.1016/j.dsx.2024.103110 39213690

[21] C. Roderburg , S. Loosen , K. Kostev , M. Demir , M. S. Joerdens , and T. Luedde , “Nonalcoholic Fatty Liver Disease Is Associated With a Higher Incidence of Coeliac Disease,” European Journal of Gastroenterology & Hepatology 34, no. 3 (2022): 328–331. 34138765 10.1097/MEG.0000000000002234

[22] M. T. Bardella , L. Valenti , C. Pagliari , et al., “Searching for Coeliac Disease in Patients With Non‐Alcoholic Fatty Liver Disease,” Digestive and Liver Disease 36, no. 5 (2004): 333–336. 15191202 10.1016/j.dld.2004.01.012

[23] J. L. Narciso‐Schiavon and L. L. Schiavon , “Fatty Liver and Celiac Disease: Why Worry?,” World Journal of Hepatology 15, no. 5 (2023): 666–674. 37305374 10.4254/wjh.v15.i5.666 PMC10251279

[24] A. Rispo , N. Imperatore , M. Guarino , et al., “Metabolic‐Associated Fatty Liver Disease (MAFLD) in Coeliac Disease,” Liver International 41, no. 4 (2021): 788–798. 33319459 10.1111/liv.14767

[25] S. Kamal , K. K. Aldossari , D. Ghoraba , et al., “Clinicopathological and Immunological Characteristics and Outcome of Concomitant Coeliac Disease and Non‐Alcoholic Fatty Liver Disease in Adults: A Large Prospective Longitudinal Study,” BMJ Open Gastroenterology 5, no. 1 (2018): e000150. 10.1136/bmjgast-2017-000150 PMC5808634 29503733

[26] M. J. Page , J. E. McKenziem , P. M. Bossuyt , et al., “The PRISMA 2020 Statement: An Updated Guideline for Reporting Systematic Reviews,” BMJ 372: n71. 10.1136/bmj.n71 PMC8005924 33782057

[27] Institute TJB , “Checklist for Cohort Studies 2017,” https://jbi.global/sites/default/files/2019‐05/JBI_Critical_Appraisal‐Checklist_for_Cohort_Studies2017_0.pdf.

[28] Institute TJB , “Ckecklist for Case Control Studies 2017,” https://jbi.global/sites/default/files/2019‐05/JBI_Critical_Appraisal‐Checklist_for_Case_Control_Studies2017_0.pdf.

[29] Institute JB , “Checklist for Analytical Cross Sectional Studies 2017,” https://jbi.global/sites/default/files/2019‐05/JBI_Critical_Appraisal‐Checklist_for_Analytical_Cross_Sectional_Studies2017_0.pdf.

[30] V. Nehra , P. Angulo , A. L. Buchman , and K. D. Lindor , “Nutritional and Metabolic Considerations in the Etiology of Nonalcoholic Steatohepatitis,” Digestive Diseases and Sciences 46, no. 11 (2001): 2347–2352. 11713934 10.1023/a:1012338828418

[31] J. Wakim‐Fleming , M. R. Pagadala , A. J. McCullough , et al., “Prevalence of Celiac Disease in Cirrhosis and Outcome of Cirrhosis on a Gluten Free Diet: A Prospective Study,” Journal of Hepatology 61, no. 3 (2014): 558–563. 24842303 10.1016/j.jhep.2014.05.020

[32] A. Renno , Y. Abdel‐Aziz , Y. Alastal , et al., “The Association Between Obstructive Sleep Apnea and Non‐Alcoholic Steatohepatitis: A Retrospective Nationwide Inpatient Sample Analysis,” Journal of Clinical and Experimental Hepatology 7, no. 1 (2021): 25–29. 10.5114/ceh.2021.104488 PMC8122103 34027112

[33] A. Bakhshipour , M. A. Kaykhaei , N. Moulaei , and M. A. Mashhadi , “Prevalence of Coeliac Disease in Patients With Non‐Alcoholic Fatty Liver Disease,” Arab Journal of Gastroenterology 14, no. 3 (2013): 113–115. 24206739 10.1016/j.ajg.2013.08.001

[34] A. R. Rahimi , N. E. Daryani , H. Ghofrani , et al., “The Prevalence of Celiac Disease Among Patients With Non‐Alcoholic Fatty Liver Disease in Iran,” Turkish Journal of Gastroenterology 22, no. 3 (2011): 300–304. 10.4318/tjg.2011.0216 21805421

[35] K. Callichurn , L. Cvetkovic , A. Therrien , C. Vincent , P. O. Hétu , and M. Bouin , “Prevalence of Celiac Disease in Patients With Primary Biliary Cholangitis,” Journal of the Canadian Association of Gastroenterology 4, no. 1 (2021): 44–47. 33644676 10.1093/jcag/gwz039 PMC7898370

[36] P. Drastich , E. Honsová , A. Lodererová , et al., “Celiac Disease Markers in Patients With Liver Diseases: A Single Center Large Scale Screening Study,” World Journal of Gastroenterology 18, no. 43 (2012): 6255–6262. 23180946 10.3748/wjg.v18.i43.6255 PMC3501774

[37] O. Lo Iacono , S. Petta , G. Venezia , et al., “Anti‐Tissue Transglutaminase Antibodies in Patients With Abnormal Liver Tests: Is It Always Coeliac Disease?,” American Journal of Gastroenterology 100, no. 11 (2005): 2472–2477. 16279902 10.1111/j.1572-0241.2005.00244.x

[38] S. Gatti , A. Rubio‐Tapia , G. Makharia , and C. Catassi , “Patient and Community Health Global Burden in a World With More Celiac Disease,” Gastroenterology 167, no. 1 (2024): 23–33. 38309629 10.1053/j.gastro.2024.01.035

[39] O. L. Iacono , S. Petta , G. Venezia , et al., “Anti‐Tissue Transglutaminase Antibodies in Patients With Abnormal Liver Tests: Is It Always Coeliac Disease?,” American Journal of Gastroenterology 100, no. 11 (2005): 2472–2477. 16279902 10.1111/j.1572-0241.2005.00244.x

[40] P. Singh , A. Arora , T. A. Strand , et al., “Global Prevalence of Celiac Disease: Systematic Review and Meta‐Analysis,” Clinical Gastroenterology and Hepatology 16, no. 6 (2018): 823–36.e2. 29551598 10.1016/j.cgh.2017.06.037

[41] A. Franzese , M. P. Iannucci , G. Valerio , et al., “Atypical Celiac Disease Presenting as Obesity‐Related Liver Dysfunction,” Journal of Pediatric Gastroenterology and Nutrition 33, no. 3 (2001): 329–332. 11593131 10.1097/00005176-200109000-00019

[42] L. Abenavoli , N. Milic , A. De Lorenzo , and F. Luzza , “A Pathogenetic Link Between Non‐Alcoholic Fatty Liver Disease and Celiac Disease,” Endocrine 43 (2013): 65–67. 22740094 10.1007/s12020-012-9731-y

[43] L. Miele , V. Valenza , G. La Torre , et al., “Increased Intestinal Permeability and Tight Junction Alterations in Nonalcoholic Fatty Liver Disease,” Hepatology 49, no. 6 (2009): 1877–1887. 19291785 10.1002/hep.22848

[44] F. Marciano , M. Savoia , and P. Vajro , “Celiac Disease‐Related Hepatic Injury: Insights Into Associated Conditions and Underlying Pathomechanisms,” Digestive and Liver Disease 48, no. 2 (2016): 112–119. 26711682 10.1016/j.dld.2015.11.013

[45] H. J. Freeman , “Hepatic Manifestations of Celiac Disease,” Clinical and Experimental Gastroenterology 33–9 (2010): 33. 10.2147/ceg.s7556 PMC3108670 21694844

[46] D. Acosta and D. G. Wenzel , “Injury Produced by Free Fatty Acids to Lysosomes and Mitochondria in Cultured Heart Muscle and Endothelial Cells,” Atherosclerosis 20, no. 3 (1974): 417–426. 4429616 10.1016/0021-9150(74)90023-9

[47] A. Wigg , I. Roberts‐Thomson , R. Dymock , P. McCarthy , R. Grose , and A. Cummins , “The Role of Small Intestinal Bacterial Overgrowth, Intestinal Permeability, Endotoxaemia, and Tumour Necrosis Factor α in the Pathogenesis of Non‐Alcoholic Steatohepatitis,” Gut 48, no. 2 (2001): 206–211. 11156641 10.1136/gut.48.2.206 PMC1728215

[48] M. Mouzaki , E. M. Comelli , B. M. Arendt , et al., “Intestinal Microbiota in Patients With Nonalcoholic Fatty Liver Disease,” Hepatology 58, no. 1 (2013): 120–127. 23401313 10.1002/hep.26319

[49] O. James and C. Day , “Non‐Alcoholic Steatohepatitis: Another Disease of Affluence,” Lancet 353, no. 9165 (1999): 1634–1636. 10335777 10.1016/S0140-6736(99)00163-4

[50] L. Valenti , P. Dongiovanni , A. Fracanzani , et al., “Increased Susceptibility to Nonalcoholic Fatty Liver Disease in Heterozygotes for the Mutation Responsible for Hereditary Hemochromatosis,” Digestive and Liver Disease 35, no. 3 (2003): 172–178. 12779071 10.1016/s1590-8658(03)00025-2

[51] H. Tilg and A. M. Diehl , “Cytokines in Alcoholic and Nonalcoholic Steatohepatitis,” New England Journal of Medicine 343, no. 20 (2000): 1467–1476. 11078773 10.1056/NEJM200011163432007

[52] Y. Sahin , “Celiac Disease in Children: A Review of the Literature,” World Journal of Clinical Pediatrics 10, no. 4 (2021): 53–71. 34316439 10.5409/wjcp.v10.i4.53 PMC8290992

[53] P. Mariani , M. G. Viti , M. Montouri , et al., “The Gluten‐Free Diet: A Nutritional Risk Factor for Adolescents With Celiac Disease?,” Journal of Pediatric Gastroenterology and Nutrition 27, no. 5 (1998): 519–523. 9822315 10.1097/00005176-199811000-00004

[54] R. Tortora , P. Capone , G. De Stefano , et al., “Metabolic Syndrome in Patients With Coeliac Disease on a Gluten‐Free Diet,” Alimentary Pharmacology & Therapeutics 41, no. 4 (2015): 352–359. 25581084 10.1111/apt.13062

[55] J. West , R. Logan , T. Card , C. Smith , and R. Hubbard , “Risk of Vascular Disease in Adults With Diagnosed Coeliac Disease: A Population‐Based Study,” Alimentary Pharmacology & Therapeutics 20, no. 1 (2004): 73–79. 15225173 10.1111/j.1365-2036.2004.02008.x

[56] F. Tovoli , G. Negrini , R. Farì , et al., “Increased Risk of Nonalcoholic Fatty Liver Disease in Patients With Coeliac Disease on a Gluten‐Free Diet: Beyond Traditional Metabolic Factors,” Alimentary Pharmacology & Therapeutics 48, no. 5 (2018): 538–546. 29984415 10.1111/apt.14910

[57] S. Q. Yang , H. Z. Lin , M. D. Lane , M. Clemens , and A. M. Diehl , “Obesity Increases Sensitivity to Endotoxin Liver Injury: Implications for the Pathogenesis of Steatohepatitis,” National Academy of Sciences of the United States of America 94, no. 6 (1997): 2557–2562. 10.1073/pnas.94.6.2557 PMC20127 9122234

[58] C. P. Day and O. F. James , Steatohepatitis: A Tale of Two “Hits”? (Elsevier, 1998), 842–845. 10.1016/s0016-5085(98)70599-2 9547102

[59] P. A. Baeuerle and T. Henkel , “Function and Activation of NF‐Kappa B in the Immune System,” Annual Review of Immunology 12 (1994): 141–179. 10.1146/annurev.iy.12.040194.001041 8011280

[60] K. S. Lee , M. Buck , K. Houglum , and M. Chojkier , “Activation of Hepatic Stellate Cells by TGF Alpha and Collagen Type I Is Mediated by Oxidative Stress Through c‐Myb Expression,” Journal of Clinical Investigation 96, no. 5 (1995): 2461–2468. 7593635 10.1172/JCI118304 PMC185899

[61] P. Bedossa , K. Houglum , C. Trautwein , A. Holstege , and M. Chojkier , “Stimulation of Collagen α1 (I) Gene Expression Is Associated With Lipid Peroxidation in Hepatocellular Injury: A Link to Tissue Fibrosis,” Hepatology 19, no. 5 (1994): 1262–1271. 8175151

[62] L. Valenti , A. L. Fracanzani , P. Dongiovanni , et al., “Tumor Necrosis Factor α Promoter Polymorphisms and Insulin Resistance in Nonalcoholic Fatty Liver Disease,” Gastroenterology 122, no. 2 (2002): 274–280. 11832442 10.1053/gast.2002.31065

