# Supplementary material for: Prevalence and Risk Factors of Celiac Disease in Patients With Non‐Alcoholic Fatty Liver Disease: A Meta‐Analysis
Source: JGH Open. 2026 Jun 22;10(6):e70434. doi: 10.1002/jgh3.70434 (PMC13287081; doi:10.1002/jgh3.70434)
Supplement: Supplementary file 1 — Table S1: Provides the complete search strategies used across all databases. Table S2: Summarizes the risk‐of‐bias assessment of the included studies according to the Joanna Briggs Institute (JBI) criteria. [file JGH3-10-e70434-s001.docx]

**Table S1: Search strategy and keywords**

| Search query | Keywords (searched through titles, abstracts, medical subject heading (MeSH), and general keywords) |
| --- | --- |
| PubMed | (((((Metabolic-Associated Steatotic Liver Disease[Title/Abstract]) OR (MASLD[Title/Abstract])) OR (MAFLD[Title/Abstract])) OR (Metabolic Dysfunction-associated Fatty Liver Disease[Title/Abstract])) OR ("Non-alcoholic Fatty Liver Disease"[Mesh])) AND (((((("Celiac Disease"[Mesh]) OR ("Coeliac Disease"[Title/Abstract])) OR ("Gluten-Sensitive Enteropathy"[Title/Abstract])) OR ("Celiac Sprue"[Title/Abstract])) OR ("Celiac Disease"[Title/Abstract])) OR ("Gluten Intolerance"[Title/Abstract])) |
| Embase | ("Non alcoholic Fatty Liver Disease" OR "NAFLD" OR "Nonalcoholic Fatty Liver Disease" OR "Fatty Liver, Nonalcoholic" OR "Fatty Livers, Nonalcoholic" OR "Liver, Nonalcoholic Fatty" OR "Livers, Nonalcoholic Fatty" OR "Nonalcoholic Fatty Liver" OR "Nonalcoholic Fatty Livers" OR "Nonalcoholic Steatohepatitis" OR "Nonalcoholic Steatohepatitides" OR "Steatohepatitides, Nonalcoholic" OR "Steatohepatitis, Nonalcoholic" OR "Metabolic Dysfunction-associated Fatty Liver Disease" OR "MASLD" OR "MAFLD" OR " Metabolic Dysfunction-associated Fatty Liver Disease") and ("Celiac Disease" OR "Disease, Celiac" OR "Gluten Enteropathy" OR "Enteropathies, Gluten" OR "Enteropathy, Gluten" OR "Gluten Enteropathies" OR "Gluten-Sensitive Enteropathy" OR "Enteropathies, Gluten-Sensitive" OR "Enteropathy, Gluten-Sensitive" OR "Gluten-Sensitive Enteropathies" OR "Gluten Sensitive Enteropathy" OR "Celiac Sprue" OR "Sprue, Celiac" OR "Sprue, Nontropical" OR "Nontropical Sprue" OR "Sprue") |
| Scopus | TITLE-ABS-KEY ( "Non alcoholic Fatty Liver Disease" OR "NAFLD" OR "Nonalcoholic Fatty Liver Disease" OR "Fatty Liver, Nonalcoholic" OR "Fatty Livers, Nonalcoholic" OR "Liver, Nonalcoholic Fatty" OR "Livers, Nonalcoholic Fatty" OR "Nonalcoholic Fatty Liver" OR "Nonalcoholic Fatty Livers" OR "Nonalcoholic Steatohepatitis" OR "Nonalcoholic Steatohepatitides" OR "Steatohepatitides, Nonalcoholic" OR "Steatohepatitis, Nonalcoholic" OR "Metabolic Dysfunction-associated Fatty Liver Disease" OR "MASLD" OR "MAFLD" OR " Metabolic Dysfunction-associated Fatty Liver Disease" ) AND TITLE-ABS-KEY ("Celiac Disease" OR "Disease, Celiac" OR "Gluten Enteropathy" OR "Enteropathies, Gluten" OR "Enteropathy, Gluten" OR "Gluten Enteropathies" OR "Gluten-Sensitive Enteropathy" OR "Enteropathies, Gluten-Sensitive" OR "Enteropathy, Gluten-Sensitive" OR "Gluten-Sensitive Enteropathies" OR "Gluten Sensitive Enteropathy" OR "Celiac Sprue" OR "Sprue, Celiac" OR "Sprue, Nontropical" OR "Nontropical Sprue" OR "Sprue") |
| ISI | (( "Non alcoholic Fatty Liver Disease" OR "NAFLD" OR "Nonalcoholic Fatty Liver Disease" OR "Fatty Liver, Nonalcoholic" OR "Fatty Livers, Nonalcoholic" OR "Liver, Nonalcoholic Fatty" OR "Livers, Nonalcoholic Fatty" OR "Nonalcoholic Fatty Liver" OR "Nonalcoholic Fatty Livers" OR "Nonalcoholic Steatohepatitis" OR "Nonalcoholic Steatohepatitides" OR "Steatohepatitides, Nonalcoholic" OR "Steatohepatitis, Nonalcoholic" OR "Metabolic Dysfunction-associated Fatty Liver Disease" OR "MASLD" OR "MAFLD" OR " Metabolic Dysfunction-associated Fatty Liver Disease" ) AND ("Celiac Disease" OR "Disease, Celiac" OR "Gluten Enteropathy" OR "Enteropathies, Gluten" OR "Enteropathy, Gluten" OR "Gluten Enteropathies" OR "Gluten-Sensitive Enteropathy" OR "Enteropathies, Gluten-Sensitive" OR "Enteropathy, Gluten-Sensitive" OR "Gluten-Sensitive Enteropathies" OR "Gluten Sensitive Enteropathy" OR "Celiac Sprue" OR "Sprue, Celiac" OR "Sprue, Nontropical" OR "Nontropical Sprue" OR "Sprue")) |

**TableS2:** Risk of bias assessment of included studies based on the Joanna Briggs Institute (JBI) tool

| Checklist Questions | Bakhshipour, 2013 | Callichurn, 2021 | Iacono, 2005 | Kamal, 2018 | Renno, 2021 | Rahimi, 2011 | Drastich, 2012 | Nehra, 2001 | Wakim-Fleming, 2014 |
| --- | --- | --- | --- | --- | --- | --- | --- | --- | --- |
| Were the criteria for inclusion in the sample clearly defined? | Yes | Yes | Yes | Yes | Yes | Yes | Yes | Yes | Yes |
| Were the study subjects and the setting described in detail? | Yes | No | Yes | Yes | No | Yes | No | Yes | No |
| Was the exposure measured in a valid and reliable way? | Yes | Yes | Yes | Yes | Yes | Yes | Unclear | Yes | Yes |
| Were objective, standard criteria used for measurement of the condition? | Yes | Yes | Yes | Yes | Unclear | Yes | Yes | No | Yes |
| Were, confounding factors identified? | Yes | No | Unclear | Yes | No | Yes | No | No | No |
| Were strategies to deal with confounding factors stated? | Yes | No | No | Yes | No | Yes | No | No | No |
| Were the outcomes measured in a valid and reliable way? | Yes | Yes | Yes | Yes | Yes | Yes | Yes | Yes | Yes |
| Was appropriate statistical analysis used? | Yes | Yes | Yes | Yes | Yes | Yes | Yes | Yes | Yes |
